# Spatiotemporal Dynamics of Argument Retrieval and Reordering: An fMRI and EEG Study on Sentence Processing

**DOI:** 10.3389/fpsyg.2012.00523

**Published:** 2012-12-11

**Authors:** Lars Meyer, Jonas Obleser, Stefan J. Kiebel, Angela D. Friederici

**Affiliations:** ^1^Department of Neuropsychology, Max Planck Institute for Human Cognitive and Brain SciencesLeipzig, Germany; ^2^Max Planck Research Group “Auditory Cognition,” Max Planck Institute for Human Cognitive and Brain SciencesLeipzig, Germany; ^3^Department of Neurology, Max Planck Institute for Human Cognitive and Brain SciencesLeipzig, Germany

**Keywords:** working memory, syntax, inferior frontal gyrus, inferior parietal cortex, argument–verb dependency, source localization, dipole time course

## Abstract

In sentence processing, it is still unclear how the neural language network successfully establishes argument–verb dependencies in its spatiotemporal neuronal dynamics. Previous work has suggested that the establishment of subject–verb and object–verb dependencies requires argument retrieval from working memory, and that dependency establishment in object-first sentences additionally necessitates argument reordering. We examine the spatiotemporal neuronal dynamics of the brain regions that subserve these sub-processes by crossing an argument reordering factor (i.e., subject-first versus object-first sentences) with an argument retrieval factor (i.e., short versus long argument–verb dependencies) in German. Using functional magnetic resonance imaging (fMRI), we found that reordering demands focally activate the left pars opercularis (Broca’s area), while storage and retrieval demands activated left temporo-parietal (TP) regions. In addition, when analyzing the time course of fMRI-informed equivalent current dipole sources in the EEG at the subcategorizing verb, we found that activity in the TP-region occurs relatively early (40–180 ms), followed by activity in Broca’s area (300–500 ms). These findings were matched by topographical correlation analyses of fMRI activations in EEG sensor space, showing that, in the scalp potential, TP-region activity surfaces as an early positivity and IFG activity as a later positivity in the scalp potential. These results provide fine-grained evidence for spatiotemporally separable sub-processes of argument retrieval and reordering in sentence processing.

## Introduction

Argument–verb dependencies have been one of the most fruitful fields in studying the cognitive architecture of sentence processing. Following initial discoveries that concurrent working memory load decreases reading-comprehension performance (Baddeley and Hitch, 1974), it was established that working memory is fundamental to sentence processing (Wingfield and Butterworth, 1984; Just and Carpenter, 1992). Specifically, King and Just’s (1991) work indicated that an individual’s working memory capacity in part determines the ability to store subject and objects until they can be retrieved at the main verb of a sentence. This entails that argument storage and retrieval are crucial roles of working memory during sentence processing.

In line with this role, event-related-brain-potential (ERP) studies isolated sustained negative ERPs for object-first as compared to subject-first sentences (Kluender and Kutas, 1993; Fiebach et al., 2001, 2002; Felser et al., 2003; Ueno and Kluender, 2003; Phillips et al., 2005). These studies directly compared object-first and subject-first sentences, not isolating the independent influence of argument–verb distance on storage load. In a recent study (Meyer et al., 2012), we directly compared short and long argument–verb dependencies irrespective of the relative order of subject and object. The result showed increased alpha-range brain oscillations during argument storage across an increasing argument–verb distance; during argument retrieval at the subcategorizing verb, both subjects and objects elicited a beta-band burst, potentially indexing increased argument retrieval demands (Gibson, 1998). This is in line with cross-modal-priming studies that have found argument-priming effects at subcategorizing verbs (Tanenhaus et al., 1985; McElree et al., 2003; Van Dyke, 2007), both for subjects and objects (Nicol, 1993; Osterhout and Swinney, 1993).

The proposal that argument storage and retrieval are common to subjects and objects is in line with the suggestion that dependency length increases retrieval demands due to decreasing activation of the argument in working memory (Gibson, 1998; Gordon et al., 2001; Lewis et al., 2006). The above ERP and priming evidence is not incompatible with proposals that dependency distance may also facilitate verb processing in verb-final sentences (Babyonyshev and Gibson, 1999; Konieczny, 2000; Vasishth, 2003; Levy, 2008; Nakatani and Gibson, 2008), based on factors such as verb–argument pre-activation (Friederici and Frisch, 2000; Kamide et al., 2000, 2003; Tsuzuki et al., 2004).

The other important sentential process under consideration, argument reordering, is conceptualized here as an executive process on the contents of working memory–-corresponding to the establishment of the underlying argument order from a sentence’s incoming argument order (Chomsky, 1955; Fodor, 1978). Early psycholinguistic research suggests that language-specific argument orders are accessed during verb comprehension, and that the mismatch between incoming order and language-specific order increases processing load at subcategorizing verbs (Rösler et al., 1998; Grodner and Gibson, 2005): for instance, grammaticality judgment is slowed down when transitive verbs are not followed by their object, but by a prepositional phrase (Clifton et al., 1984); furthermore, the preference to interpret a post-verbal noun phrase as a transitive verb’s direct object is so strong as to erroneously hinder this noun phrase’s interpretation as the subject of a subsequent sentence (Trueswell et al., 1993). For English, such effects may be explained in terms of activation decay as well (Gibson, 1998): often-queried English object-relative clauses change the argument order, but collaterally increase the argument–verb distance over subject-relative clauses. German, however, allows for changing the argument order without changing the argument–verb distance, ruling out potential decay explanations of argument order effects in German. In line with this, Konieczny and Döring (2003) have shown that an increased number of verb-adjacent arguments in German sentences differentially decreases processing load at sentence-final main verbs, even if argument–verb distance is controlled for.

Offering a potential reconciliation of the distance-based and reordering-related proposals, our recent self-paced-reading study (Meyer et al., 2010) fully crossed argument–verb distance and argument order, extending Konieczny and Döring’s (2003) results: German object-first argument orders were found to increase sentence-final processing load as compared to subject-first argument orders. The data also showed that long argument–verb distance increases processing load only for subject-first sentences, but decreases processing load for object-first sentences, which can be explained by a dynamic interplay of working memory decay (Gibson, 1998) and argument order-facilitated verb prediction (Levy, 2008).

While there is the possibility that argument–verb distance and argument order interact behaviorally, neuroimaging work has found manipulations of argument order and argument–verb distance to activate very distinct brain regions: In a recent functional-magnetic-resonance-imaging (fMRI) study, we also orthogonally manipulated dependency length and argument order (Meyer et al., 2012b). We found significant activity in the left temporo-parietal (TP) region for long as compared to short argument–verb distances, while the left inferior frontal gyrus (IFG) was significantly active for object-first as compared to subject-first argument orders. No interaction was found on the neural level. A role of the TP-region in storage is in line with previous imaging research (Grossman et al., 2002; Novais-Santos et al., 2007), and the region’s role in retrieval has been suggested as well (Henson et al., 1999; Buchsbaum et al., 2011). The IFG responsivity to increasing argument reordering demands is in line with cross-linguistic data on argument order processing (Ben-Shachar et al., 2003; Friederici et al., 2006; Kinno et al., 2008) and sequencing tasks outside of the sentence processing domain (Gerton et al., 2004; Clerget et al., 2011; Makuuchi et al., 2012). From a cognitive-neuroscience perspective, the brain data gives reason to conceptualize reordering as an executive operation on working memory contents (Wingfield and Butterworth, 1984). Such a conceptualization takes into account previous reports on the neural independence of word-order-related processes and working memory proper during sentence processing (Caplan and Waters, 1999; Caplan et al., 2000; Fedorenko et al., 2011). While the behavioral predictions of this mechanism may parallel those of frequency-based processing theories that describe reordering-related processing load by the decreased corpus frequency of reordering-intensive constructions (Levy, 2008), there is previous evidence that the neural sensitivity to argument reordering demands is independent of structural frequencies (Bornkessel et al., 2002).

In sum, storage-and-retrieval-related brain activity in the TP-region may be linked to Gibson’s (1998) conceptualization of retrieval difficulty as induced by activation decay. Argument order-related brain activity in the IFG, on the other hand, may be linked to an executive reordering processes (Meyer et al., 2012b), potentially mirrored in corpus-derived syntactic-frequency data (Levy, 2008). The important question under investigation here is how the proposed processes of argument retrieval and reordering map onto the spatiotemporal neuronal dynamics of this underlying neuroanatomical network. We used a previously implemented paradigm that orthogonally manipulated argument–verb distance and argument order in German sentences. To perform the current combined fMRI–EEG analyses, previously acquired fMRI (Meyer et al., 2012b) and EEG data (Meyer et al., 2012) were re-analyzed from a sub-sample of 14 participants who had participated in both of these studies. By using a combined analysis, we can, in principle, map temporal EEG dynamics to spatially precisely defined regions derived from the fMRI results.

As outlined above, we expected that argument retrieval at subcategorizing verbs is harder for long as compared to short argument–verb distances due to increased memory decay (Gibson, 1998, 2000; Baddeley, 2012). Alternatively, argument retrieval might be easy due to increased verb anticipability (Konieczny, 2000; Levy, 2008). The decay hypothesis predicts long argument–verb distances to increase TP-region brain activity in response to the subcategorizing verb, leading to an increased response in the EEG source time course. Since retrieval has been related to the P300 range, this effect should start early (Ergen et al., 2012; see Discussion) and may be initiated prior to the subcategorizing verb (Fiebach et al., 2001). The anticipability hypothesis predicts that long argument–verb distances decrease TP-region brain activity, leading to a decreased response in EEG source time course. Predictability has been related to the N400-range, so this effect should start later (Van Petten, 1993). On the EEG sensor-level, the first hypothesis predicts an increased correlation of the ERP over posterior sensors with the TP-region fMRI effect in the in a P300 time window. The second hypothesis predicts a decreased correlation in a N400 time window.

Here, we hypothesized that increased argument reordering demands (object-first as compared to subject-first argument orders) subsequently increase brain responses in the left IFG during the processing of subcategorizing verbs. This should be reflected by an increased response in the IFG dipole time course for object-first argument orders as well as an increased correlation of the ERP over frontal sensors with the fMRI effect in the IFG region. Because previous research has found argument reordering demands to drive ERP effects in the P600 range (cf. Friederici et al., 2002; see Discussion), we expected these effects to occur in the P600 range. To test our hypotheses, we used two complementary analysis techniques: firstly, we reconstructed the time course of the TP and IFG regions of interest from ERP data to test the hypothesis about the temporal sequence of brain activation due to reordering and retrieval. Secondly, we correlated task-specific fMRI activations of these two regions with the ERPs at the sensor-level.

## Materials and Methods

### Participants

Data of 14 participants [mean age 26.7 years, standard deviation (SD) 3.5 years, six females, all German native speakers] were analyzed. These data were taken from two larger studies of 24 participants (fMRI study) and 36 participants [electroencephalography (EEG) study], where we chose those participants who had taken part in both experiments (in counterbalanced order; minimum time between sessions was 53 days, maximum 160 days). Participants had been matched for their reading span being in the range between 2.5 and 4.5 (mean 3.8, SD 0.7) according to an abridged version of the reading span test (Daneman and Carpenter, 1980). All participants were right-handed as assessed by an abridged version of the Edinburgh Inventory (Oldfield, 1971), reported no neurological or hearing deficits, and had normal or corrected to normal vision. Participants were paid €14 for participation in the fMRI study and €17.50 for participation in the EEG study. Written informed consent was obtained from all participants. All procedures received ethical approval by the local ethics committee (University of Leipzig).

### Materials

**Figure 1:**
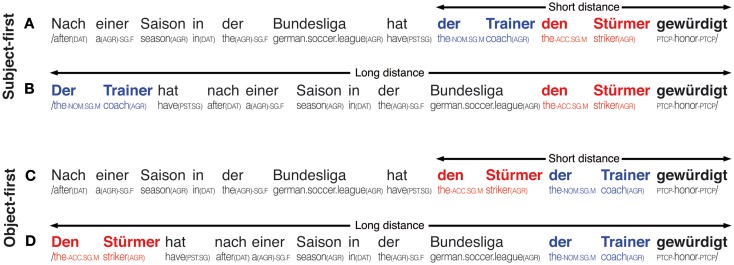
**Overview of stimulus sentences; upper panel shows subject-first argument orders in the short (A) and long (B) distance argument–verb distance variants, lower panel shows object-first argument orders in the short (C) and long (D) argument–verb distance variants; individual glosses are provided below each example**. The subject of each sentence is marked in bold blue, each sentence’s object is marked in bold red, the subcategorizing verb is marked in bold black font. All sentences translate to *After a season in the German soccer league, the coach honored the striker*.

German sentences allowing for an orthogonal manipulation of one factor solely affecting reordering and one factor solely affecting retrieval were constructed. Accordingly, our 2 × 2 factorial design crossed the factors reordering (subject-first versus object-first) and retrieval (short versus long), as shown in Figure 1.

In the first and second condition (A and B), the argument order is subject-first, while the argument–verb distance is either short (A) or long (B). The third and fourth conditions (C and D) apply the short (C) and long (D) distance variants to an object-first argument order. Across the levels of the reordering factor, the long variants (B and D) increase the retrieval demands over their respective short counterparts (A and C) by lengthening the necessary retention interval for the critical information of the subject or object noun phrase. Crucially, retrieval demands are identical for the two short and the two long conditions, respectively (i.e., in the short conditions, one phrase intervenes between the first argument and the main verb, whereas in the long conditions, four phrases intervene)–-and independent of the relative order of arguments. The absolute position of the main verb–-where the parser can determine verb transitivity and argument order–-is kept constant across conditions to avoid the potential confound that processing speed increases toward sentence ending (Ferreira and Henderson, 1993). In order to avoid a possible sentence-final processing slowdown due to increasing propositional and semantic load (Friedman et al., 1975), we chose to add a conjunct clause (not shown in Figure 1; e.g., “und die Entwicklung bestätigt,” translating to *and confirmed the development*. for the examples in Figure 1), which was identical across all four experimental conditions of a set. To also avoid any influence of frequency and semantic coherence on the experimental manipulations (Van Petten, 1993; Levy, 2008), a position-wise lemma frequency and syllable count matching using the CELEX database (Baayen et al., 1995) was performed and supplemented by sentential neighborhood analyses using the Projekt Deutscher Wortschatz database (Biemann et al., 2004). Specifically, each sentence’s subject and object were balanced in length and frequency to avoid systematic confounding of the reordering manipulation.

In total, 48 sentences in the four conditions were generated, resulting in a set of 192 stimuli. For the fMRI study, 192 additional filler sentences (e.g., “Gestern hat der Leser dem Bibliothekar den Artikel zurückgegeben,” translating *Yesterday, the reader gave back the article to the librarian*) of differing ordering and retrieval demands from a previous study (Friederici et al., 2006) were used to avoid multicollinearity in the design (Andrade et al., 1999). To fit these sentences to the experimental stimuli, conjunct clauses (e.g., “und die Gebühr bezahlt,” translating *and paid the fees*.) were added as well, using the above frequency-matching procedures. For the EEG study, we chose to not use filler sentences, to keep the recording sessions as short as possible because long-lasting EEG recording sessions tend to decrease participants’ attention toward the end of the experimental run, with the amount of recording artifacts in the data increasing. As a second measure to ensure participants’ attentive engagement in the task, 25% of all trials (i.e., 48 sentences per participant) were task trials which required feedback by the participants (see below).

For the fMRI study, each participant received an individual list of 216 stimuli from the stimulus pool of 384 sentences: 144 stimulus sentences (36 per condition), 36 filler sentences, and 36 null events were drawn in a counterbalanced way using MATLAB^®^ 7.9 (The MathWorks, Inc., Natick, MA, USA) scripts. For the EEG study, each participant received the total stimulus set of 192 stimuli. As a task to maintain participants’ attention in both the fMRI and the EEG study, 16.7% (fMRI) and 25% of trials (EEG) introduced a who-did-what-to-whom yes/no comprehension question (e.g., “Hat der Trainer den Stürmer geehrt?” translating *Did the coach honor the center forward?*). The proportion of yes–correct and no–incorrect questions was balanced. Experimental items and fillers were recorded in a soundproof chamber by a trained female German speaker with a Sennheiser^®^ MKH 40 condenser microphone and a Roland^®^ CD-2 digital sound recorder. Recordings were cut and normalized in Praat (Boersma and Weenink, 2001) by the root mean square amplitude of all recordings. To avoid onset and offset artifacts, a cosine fade in and out sequence of 5 ms was attached.

### Procedure

Stimulation was performed using the software package Presentation^®^ (Neurobehavioral Systems, Inc., Albany, CA, USA). For the fMRI study, the auditory stimuli were presented using air-conduction headphones (Resonance Technology, Inc., Northridge, CA, USA). Visual stimuli were presented on a Sanyo PLC-XP50L LCD XGA mirror-projection system with a refresh rate of 100 Hz (Sanyo Electric Co., Ltd., Moriguchi, Japan), mounted onto the headcoil. In the EEG study, auditory stimuli were presented using a pair of Infinity^®^ Reference I MkII stereo speakers (Harman International Industries, Inc., Stamford, CT, USA), approximately 100 cm to the left and right front of the participants. Visual stimuli were presented using a Sony Trinitron^®^ Multiscan G220 CRT VGA monitor with a refresh rate of 75 Hz (Sony Corporation, Tokyo, Japan), approximately 70 cm in front of the participants. Across recording modalities, visual stimuli appeared in a sans-serif font in black letters against a gray background (font size 20 px).

In the fMRI study, a trial started with a fixation cross that stayed on screen for the whole trial. After a random jitter of 0, 500, 1000, or 1500 ms, an auditory stimulus started (mean length 4.9 s, SD 0.36 s). To keep the number of acquired volumes (and thus the signal-to-noise ratio) constant across conditions in spite of jittering, we inserted a silent period and an on-screen fixation cross between stimulus and trial ending to achieve a constant trial duration of 8 s. Such a sequence was either followed by the next trial or by a fixation cross using a random jitter and a subsequent visual comprehension question (16.7% of all trials). The question remained on screen for 1500 ms, and participants had been instructed initially to answer the question as quickly as possible during this time period. Visual feedback was given for 1000 ms by a green happy or red sad emoticon. To also keep the duration of the comprehension probes constant, silence, and an on-screen fixation cross were inserted, such that each comprehension probe would last 4 s. Participants were instructed to answer the comprehension questions via button press with either their left or right hand, with one hand corresponding to yes and the other to no. Response button assignment was counterbalanced across participants.

In the EEG study, a trial started with a green fixation cross of a random length between 2000 and 3500 ms (uniform distribution). After this period, the fixation cross turned red, and an auditory stimulus was presented. This extended prolog was used to avoid oculomotor artifacts, which else would threaten to decrease the signal-to-noise ratio: participants were instructed to blink only when the fixation cross was green. Either the next trial followed, or–-in 25% of trials–-a yes–no comprehension question. Participants had to answer these questions by using either their left or right hand to press one of the two buttons of a two-button response box. Response button assignment was counterbalanced across participants. Prior to comprehension questions, a green fixation cross of a random length was presented to avoid task-preparation effects during the processing of the acoustic input. To ensure participants’ comfort and avoid task artifacts (Hagoort et al., 1993), comprehension questions stayed on screen until a button press occurred. Like in the fMRI procedure, visual feedback was given after comprehension questions for 800 ms in the form of a happy green or sad red emoticon. An experimental run, consisting of 192 trials, lasted for approximately 35 min. Including preparation and electromagnetic position tracking (see below), the experiment lasted approximately 1.5 h.

In summary, the main difference between the EEG and fMRI sessions was the unconstrained task interval in the EEG as compared to the constrained task interval in the fMRI study, where the comprehension question had to be answered within 1500 ms. Further differences included the absence of filler items and null events in the EEG study as compared to the fMRI as well as the different numbers of experimental items per participant in the EEG (all 192 stimuli of the set) and fMRI studies (144 of the 192 stimuli). Furthermore, the pre-stimulus interval in the EEG study was between 2000 and 3500 ms, whereas in the fMRI study, a random jitter of 0, 500, 1000, or 1500 ms was used. The percentage of task trials was 25% in the EEG study and 16.7% in the fMRI study.

### Data acquisition

All MRI data were acquired with a three Tesla Siemens TIM TRIO scanner (Siemens Healthcare, Erlangen, Germany) and a 12-channel headcoil at the Max Planck Institute for Human Cognitive and Brain Sciences in Leipzig, Germany. T1-weighted 3D magnetization-prepared rapid gradient echo images (Mugler III and Brookeman, 1990; TA = 650 ms; TR = 1300 ms; alpha = 10°; FOV = 256 mm × 240 mm; two acquisitions; 1 mm isotropic resolution) had been previously acquired with a non-slice-selective inversion pulse followed by a single excitation of each slice, and were available for preprocessing of the functional data as well as subsequent source time course analysis of the EEG data. Functional MR data were acquired using a T2*-weighted gradient echo echo-planar-imaging sequence (data matrix 64 × 64, TR 2.0 s, continuous scanning, TE = 30 ms, flip angle = 90°, bandwidth 116 kHz, FOV 19.2 cm, in-plane resolution 3 mm × 3 mm, slice thickness 3 mm, interslice gap 1 mm, 30 horizontal slices parallel to AC-PC line, whole-brain coverage, 912 volumes), with a functional scan time of 30 min.

The EEG was recorded with a pair of Brainvision BrainAmp DC amplifiers (Brain Products GmbH, Munich, Germany) from 64 tin scalp electrodes, attached to an elastic cap (Electro-Cap International, Inc., Eaton, OH, USA) and placed at standard positions of the extended International 10–20 system. Each of the electrodes was referenced to the left mastoid, and the setup grounded to the sternum. The vertical electro-oculogram (EOG) was recorded from electrodes located above and below the left eye. The horizontal EOG was recorded from electrodes positioned at the outer canthus of each eye. The resistance of the electrodes was kept below 3 kΩ. The EEG and EOG were recorded continuously with a band-pass filter from DC to 250 Hz with a sampling rate of 500 Hz. Each recording was followed by tracking of the individual electrode positions, using a Polhemus FASTRAK^®^ electromagnetic motion tracker (Polhemus, Colchester, VT, USA).

### Data analysis

For assessment of behavioral performance in both the fMRI and EEG study, we calculated *d*′-scores (Macmillan and Creelman, 2005). Although mean percentage correct scores are reported more frequently, *d*′-scores have the advantage of eliminating participants’ response bias, i.e., the individual tendency to either press the yes–correct or no–incorrect button, and are thus a more adequate representation of participants’ performance (Macmillan and Creelman, 2005). For both the fMRI and EEG study, a one-sample *t*-test on the difference between the mean *d*′-scores and chance level performance (*d*′ = 0, i.e., 50% correct responses) was performed. A 2 (fMRI versus EEG study) × 2 (subject-first versus object-first) × 2 (short versus long) analysis of variance (ANOVA) was run on the response data to determine experiment- and condition-specific effects.

Analysis of fMRI data was performed using the SPM 8 software package (Wellcome Trust Center for Neuroimaging, University College London, London, UK; http://www.fil.ion.ucl.ac.uk/spm/). Before statistical analysis, the data were co-registered to previously acquired high-resolution 3D structural images and resampled to 3 × 3 × 3 mm^3^ voxel size. The functional time series were spatially aligned to the first image, corrected for local MR field inhomogeneities (“unwarped”), and temporally interpolated to correct for slice acquisition timing. Next, normalization to a standard MR template (gray-matter segmentation-based procedure) and smoothing using an isotropic 8 mm^3^ kernel were applied.

At the first-level, the fMRI data was analyzed based on a general linear model approach, using the canonical hemodynamic response function in the SPM8 package. Trial-specific stimulus lengths were taken into account, and fillers, silent trials and visual task trials were treated as regressors of no interest. To remove slow global signal changes, a high-pass filter of 1/100 s was used. Individual contrast estimates for the four experimental conditions were derived. The time window of interest included the subcategorizing verb, but was collapsed across the whole sentence (i.e., 2.5 TRs on average) to increase the signal-to-noise-ratio in our statistically confined 14-participant sample as compared to a verb-only epoch (see Results). The estimates were passed into a second-level within-subject ANOVA, where main effects and interactions were assessed. There were strong *a priori* hypotheses on expected neuroanatomical regions from independent work (Grossman et al., 2002; Ben-Shachar et al., 2003; Friederici et al., 2006; Novais-Santos et al., 2007; Kinno et al., 2008) and our own study on the original 24-participant sample (Meyer et al., 2012b). Since our previous study gave us clear activation peaks at *p* < 0.005 (corrected for multiple comparisons), we thresholded the statistical maps in the present study at a more lenient threshold of *p* < 0.005 (uncorrected), choosing those activations that resembled in their location and extent the original regions of interest (ROIs) identified in the full 24-participant sample.

For EEG data analysis, the Fieldtrip toolbox for EEG/MEG analysis (Oostenveld et al., 2011) was used. The raw data were high-pass filtered with a cut-off frequency of 0.03 Hz with a Hamming-windowed sixth-order two-pass Finite Impulse Response filter (Edgar et al., 2005) to remove slow electrode drifts. For each experimental trial, a 1-s main verb epoch spanning the subcategorizing verb (mean onset latency 2933 ms, SD 276 ms) was extracted from the data, including a 200-ms pre-stimulus baseline. For artifact rejection, EEG epochs were off-line re-referenced to linked mastoid electrodes, and automatic EOG and muscle artifact rejection was performed on a trial-by-channel basis. Cutoffs for the EOG and muscle artifact rejection were based on specifically band-pass-filtered, *z*-transformed distribution of all observed trial amplitudes and set at *z* = 3 and *z* = 7 (within bands of 1–14 Hz and 110–140 Hz), respectively. The routine resulted in the rejection of 16.3% of trials.

### Combined fMRI–EEG analysis in sensor and source space

For the combined fMRI–EEG analysis in sensor space, fMRI-informed topographical correlation analyses were performed. To use the current functional effects as regressors in this analysis, we extracted individual percentages of signal change for the four experimental conditions, masking the individual activation maps from the first-level analysis with the functionally defined group-level ROIs (see Results). By averaging across the respective conditions, we formed regressors for the four factor levels subject-first (short and long), object-first (short and long), short (subject-first and object-first), and long (subject-first and object-first). To reduce the amount of data, we down-sampled the ERPs and their respective baseline periods to 100 Hz. We then calculated four mean ERPs for each participant, corresponding to the four design levels extracted from the fMRI data, resulting in an individual average ERP for the subject-first (short and long), object-first (short and long), short (subject-first and object-first), and long (subject-first and object-first) conditions. The 14 individual ERPs for each of these four levels underwent across participants electrode- and sample-wise Pearson’s linear regression analyses with the fMRI-based regressors (see above), resulting in a time series of 120 (200 ms baseline period plus 1 s ERP) topographical coefficient maps for the correlation between the fMRI signal change and the ERPs for the four respective levels. To retrieve the final statistical maps for the two main effects of our design, we *z*-transformed these maps for the four levels and computed a difference map for the main effect of reordering (object-first minus subject-first) and retrieval (long minus short). The resulting difference values were divided by the difference between the standard errors, and converted to *p*-values, which underwent false-discovery rate (FDR) correction across samples and electrodes (Benjamini and Hochberg, 1995) to control for the inflated type I error risk.

For the combined fMRI–EEG analysis in source space, ERP-informed dipole time course analyses were performed in SPM8, using spatially precise fMRI priors (Daunizeau et al., 2010). First, the individual high-resolution anatomical scans were normalized to MNI space using both Fieldtrip and SPM8. After unified segmentation, individual boundary element models (BEMs, Besl and McKay, 1992) were generated, to which the individually determined electrode positions were co-registered. A leadfield matrix for each point in this volume conductor was generated using the Fieldtrip toolbox for EEG/MEG analysis (Oostenveld et al., 2011). For the source time course analysis, a Variational Bayes Equivalent Current Dipole (VB-ECD) procedure (Kiebel et al., 2008) was applied. We used subject-specific fMRI-based location priors for the IFG and TP-region (see Results) to derive source time courses. Location priors were derived by determining the Montreal Neurological Institute (MNI) coordinate of the individual statistical peak voxel inside the respective group-level ROI (see Results) where we used a first-level *t*-contrast for the respective main effect, masked for the respective ROI (i.e., object-first greater subject-first for the IFG ROI, long greater short for the TP-region ROI, see Results). Importantly, these spatial priors do only contain information about the location of an individual peak voxel, but not its functional activation strength – hence, the reconstructed source time courses are not biased by the functional activation itself (cf. Vul and Pashler, 2012). The dipoles at these prior positions were allowed to relocate freely inside a radius of 0.5 cm and to change their orientation and moment, following the analysis strategy used by Friederici et al. (2000). The VB-ECD algorithm was set to perform 10 iterations of minimizing the negative free energy *F* to fit the dipole locations and orientations to the actual sensor data across trials and conditions. The dipole locations and orientations were determined at convergence, and the individual EEG sensor data were projected onto these dipoles through the individual leadfield matrix–-resulting in final time courses of dipole moments Q_x_, Q_y_, and Q_z_ in all three spatial directions. For each ROI, the first eigenvariate from a principal component analysis on these three dipole moments was used to arrive at a single time course for each trial. Finally, time courses for each ROI and participant were averaged and *t*-tests were performed on specific time windows (see Results).

## Results

### Behavioral data

For the fMRI data, mean *d*′-score was 0.6 (SD 1.0), which was significantly different from chance [*t*(13) = 2.17, *p* < 0.05]. For the EEG data, mean *d*′-score was 2.8 (SD 0.5), which was significantly different from chance [*t*(13) = 21.05, *p* < 0.001]. The ANOVA on the condition-specific scores for the fMRI and EEG experiment showed a main effect of experiment [fMRI versus EEG; *F*(2,13) = 116.60, *p* < 0.001], with no other main effects or interactions present.

### Combined analyses of fMRI and ERP data

**Figure 2:**
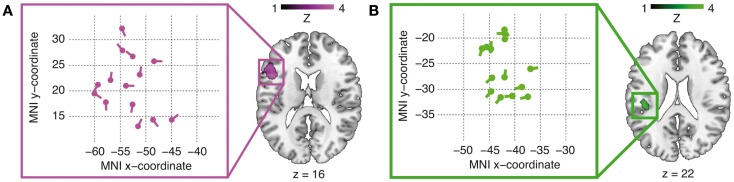
**Brain activations for the main effects of argument order [magenta cluster in (A)] and argument–verb distance [green cluster in (B)]**. Activations are thresholded at *p* < 0.005 at a minimum cluster size of 20 suprathreshold voxels. The respective coordinate systems (left) illustrate the distribution of the individual dipole locations and orientations inside the IFG **(A)** and TP-region **(B)** group activation clusters in the axial plane after relocation.

As functionally defined spatial priors for the combined fMRI–EEG analyses, two main activation foci were determined from the fMRI data, applying stimulus functions using the duration of the whole sentences (see above), shown in Figure 2.

A test for the main effect of reordering (object-first sentences leading to more activation than subject-first sentences) yielded a peak in the left IFG (group-level peak at *x* = −45, *y* = 14, *z* = 16, peak *Z*-score 3.21, peak-level *p* < 0.001, cluster size 127 voxels), shown in panel A of Figure 2. The activation focus for the main effect of retrieval (long argument–verb distances leading to stronger activation than short argument–verb distances) was obtained inside the left TP-region, more specifically the Rolandic operculum (group-level peak at *x* = −42, *y* = −25, *z* = 22, peak *Z*-score 3.38, peak-level *p* < 0.001, cluster size 23 voxels), shown in panel B of Figure 2. Both of these clusters were the most significant activations in the respective contrasts, with some other activations shown in Table 1. Note also that a confirmatory analysis on short-duration verb-onset epochs (as opposed to the whole sentence epoch, see Data Analysis) yielded identical peak voxels (IFG: *x* = −45, *y* = 14, *z* = 16, peak *Z*-score 3.85, peak-level *p* < 0.001; TP-region: *x* = −42, *y* = −25, *z* = 22, peak *Z*-score 3.94, peak-level *p* < 0.001). Because no significant interactions were found at the whole-brain level, we restricted our combined analyses to the main effects of reordering (object-first versus subject-first) and retrieval (long versus short). Table 1 summarizes the activations (minimal cluster size 20 voxels).

**Table 1:** List of significant clusters in the fMRI contrasts, thresholded at *p* < 0.005 and a minimum cluster extent of 20 voxels*.

Site	MNI-coordinate			Cluster size (mm^3^)	*Z*-score
	*x*	*y*	*z*	
**Main effect of reordering factor (object-first > subject-first)**					
IFG/BA 44*	−45	14	16	1143	3.21
	−60	20	13		3.14
	−51	20	22		3.13
IFG/BA 45*	−42	5	52	243	3.01
**Main effect of retrieval factor (long > short)**					
Rolandic operculum*	−42	−25	22	207	3.38
Postcentral gyrus/area 3a*	−18	−34	49	198	2.92
	−30	−31	52		2.61
	−27	−40	46		2.60

*According to Eickhoff et al. (2005).

**Figure 3:**
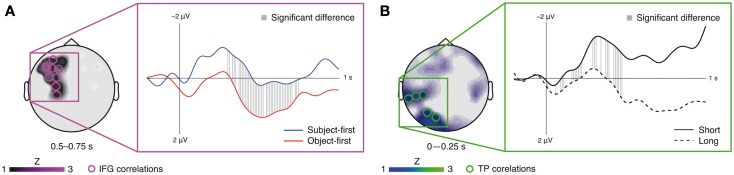
**Results of the fMRI-informed topographical correlation analysis in EEG sensor space for the IFG (A) and TP-region (B) analysis, respectively**. Circles mark EEG sensors at which significant fMRI–EEG correlation differences were obtained, whereby the topography represents the average correlation across the named time window. Corresponding waveforms show the average ERPs across these EEG sensors. Gray lines mark significant sample-wise correlation differences (*p* < 0.05, FDR-corrected).

The fMRI-informed topographical correlation analysis in EEG sensor space for the ERP at the subcategorizing verb yielded a significant (mostly) late left frontal correlation for the reordering factor (i.e., a significant difference in correlations between the object-first and subject-first sentences with the signal change in the IFG ROI). This correlation difference was present at electrode AF7 (0–250 ms), electrodes FC5, F7, AF7, FP1, FP2, and F8 (251–500 ms), electrodes FC5, F5, F7, C3, FC3, F3, AF3, AF7, and CP3 (501–750 ms), and electrode F7 (751–1000 ms). In the averaged time course over these sensors the significant difference lasts from 300 ms to 740 ms (Figure 3A; *p* < 0.05, FDR-corrected). The analogous analysis for the retrieval factor (i.e., the difference in correlations between the long and short argument–verb dependencies with the signal change in the TP-region ROI) yielded a significant mostly early left posterior correlation at electrodes CP5, TP7, TP9, PO7, and O1 (0–250 ms) and electrodes PO7 and O1 (251–500 ms), lasting from 10 ms to 470 ms in the average over these sensors (Figure 3B; *p* < 0.05, FDR-corrected).

The dipole time course analysis in MRI source space for the main verb epoch allowed for slight relocations of the fMRI priors by the VB-ECD algorithm. All average position changes were negligible (<1 mm), resulting in an average relocated IFG dipole position of *x* = −53, *y* = 21, *z* = 20 and an average relocated TP-region dipole position of *x* = −42, *y* = −26, *z* = 20 (individual dipole locations after relocation are shown in panel A and B of Figure 2). Statistical analyses of the dipole time courses (object-first and subject-first across the individual IFG dipole positions, long and short across the individual TP-region dipole positions) in two time windows defined by our prior hypotheses (300–500 ms for the IFG dipole time course, 200–300 ms for the TP-time course) showed significantly increased dipole activity in the IFG for the object-first as compared to the subject-first argument orders from 300 to 500 ms.

## Discussion

### Behavioral data

The statistical analysis on the behavioral data from our 14 participants suggests that while behavioral performance in both the fMRI and the EEG study was significantly above chance, performance in the fMRI study was significantly worse than it was in the EEG study. As a second finding, there were no condition-specific effects in either the fMRI or the EEG study. The first finding means that the participants were able to process and comprehend all four conditions of our paradigm. The difference in performance between the fMRI and the EEG study may be explained by the different task procedures: the response window in the fMRI study was strongly time-constrained, whereas in the EEG study it was not. Given participants’ ability to process our stimuli in the EEG study, we are confident that the task in the fMRI study served to keep participants’ attention directed toward the sentences, rendering the results valid. While the second finding may in principle speak in favor of a balanced design without processing difficulty confounds, it must be interpreted with caution: previous studies have found both distance-related (Gibson, 1998; Gordon et al., 2001; Lewis et al., 2006) and order-related (Rösler et al., 1998; Grodner and Gibson, 2005) processing load differences at main verbs in sentences comparable to the present stimuli. Hence, alternative explanations of the null result include a lack of statistical power in our confined sample or a lack in methodological sensitivity due to the off-line character of our experimental task.

### fMRI results and combined analyses

**Figure 4:**
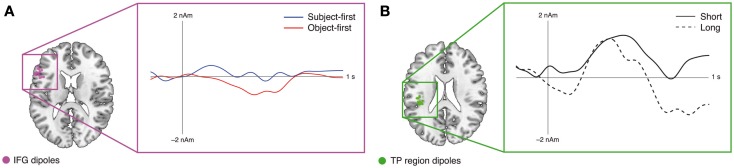
**Results of the ERP-informed dipole time course analysis in MRI source space for the IFG (A) and TP-region (B) dipoles**. Dots in the brain renderings mark the final dipole positions; the respective waveforms illustrate the reconstructed grand average dipole time courses across IFG **(A)** and TP-region **(B)** dipoles. For the TP-region, an early time window was selected after visual data inspection, showing an early difference between the short and long argument–verb distances, surfacing as an early left posterior positivity (*p* < 0.05, uncorrected). The IFG shows a later difference between the subject-first and object-first argument orders, surfacing as a late left frontal positivity (*p* < 0.05, uncorrected).

The fMRI results on the restricted sample are in line with the previously published data from the full 24-participant sample (Meyer et al., 2012b): the TP-region appears sensitive to argument retrieval demands, whereas the IFG appears sensitive to argument reordering demands. In general, this is in line with the previously suggested independence of prefrontal argument – order-related brain activity from working memory processes during sentence processing (Caplan et al., 2000; Fedorenko et al., 2011). The combined fMRI–EEG analyses at the subcategorizing verb – the main focus of the current study – suggest that IFG source activity occurs relatively late, whereas TP-region activity occurs early. While our hypothesis-based time window for the TP-region time course did not yield a significant difference between the short and long conditions, we note that visual inspection (see Figure 4B) informed us about a second, earlier time window showing a significant difference (75 samples from 35 to 180 ms). For this *post hoc*-selected time window we found *t*(13) = 2.57, *p* < 0.05 for the long as compared to the short argument–verb distances (Figure 4B). Topographical correlation analyses showed that TP-region activity surfaces in an early left posterior positivity, whereas IFG activity surfaces in a left frontal late positivity (Figure 3). Both correlation and source space results speak in favor of a plausible role of the IFG and TP-region dipoles in the generation of the observed ERP responses: based on previous reports (Friederici et al., 2006; Novais-Santos et al., 2007), we suggest that the reconstructed source time courses of the IFG and TP-region, in response to the subcategorizing verb, reflect the activation time course of the neuroanatomical substrates of early argument retrieval (TP-region) and late argument reordering (IFG) at subcategorizing verbs. Furthermore, we suggest that the sensor-level fMRI–EEG correlations mirror this functional course of retrieval and reordering. In the following, we will discuss each the TP-region and IFG results from the combined analyses, directly relating these to the associated fMRI effects.

### Early TP-region activity: Argument retrieval

The current fMRI analyses support a role of the TP-region in argument retrieval, which is plausible given previous reports of a role of the left SMG and inferior and posterior parietal regions during verbal retrieval outside of sentence processing (Henson et al., 1999; Buchsbaum et al., 2001; Ravizza et al., 2004, 2011). The posterior STG has also been found active for verbal storage during the processing of ambiguous sentences with long retention intervals for the disambiguating information (Novais-Santos et al., 2007). In addition, reduced verbal-working memory-related parietal brain activity has been suggested as a source of sentence processing difficulties in seniors (Grossman et al., 2002). From a psycholinguistic perspective, increased TP-region activity during our storage-and-retrieval-intensive conditions may mirror the increased processing load in counteracting increased memory decay (Gibson, 1998, 2000). In apparent opposition to these findings, further studies suggest an involvement of BA 45 in the prefrontal cortex in verbal working memory during sentence processing. This counts for English work that compared pronoun binding to argument–verb dependencies (Santi and Grodzinsky, 2007) or argument–verb dependencies to embedded sentences (Santi and Grodzinsky, 2010). It also counts for German work manipulating subject-argument–verb distance (Makuuchi et al., 2009), whereby the number of argument–verb dependencies varied across the levels of the distance manipulation. A second German study by Fiebach et al. (2005) found BA 45 activation, contrasting object-first sentences with an object pronoun (and a subject noun) to subject-first sentences with a subject pronoun (and an object noun). The asymmetric comparisons in the above studies may have engaged a syntactic-working memory system, distinct from the system used in other verbal tasks (Fedorenko et al., 2006; Van Dyke, 2007), and proposed to rely on BA 45 (Caplan et al., 2000). In contrast, the current paradigm kept the type of syntactic dependency constant across conditions.

The current combined fMRI–EEG analyses found that the increased TP-region activity observed in the fMRI analyses surfaces as an early left TP positivity. This matches our hypothesis that long argument–verb distances result in increased argument retrieval demands at the subcategorizing verb. The fact that an independent analysis showed TP-region activity on the source level to occur during a similar time window supports this interpretation. We suggest that the combined fMRI–EEG results provide a possible link between fMRI evidence on TP-region involvement in retrieval (Henson et al., 1999; Buchsbaum et al., 2001; Ravizza et al., 2004, 2011), ERP findings of early positive responses during retrieval (Grossberg, 1984; Donchin and Coles, 1988), and psycholinguistic work from the sentence processing domain showing increased argument retrieval demands at subcategorizing verbs during sentence processing (Gibson, 1998, 2000). Outside the sentence processing domain, there has been a previous proposal along these lines (Birbaumer et al., 1990); however, there remained a lack of fMRI–EEG studies that merit a direct connection.

This proposal is in line with previous findings: Friedman et al.’s (1975) ERP study required sentence-final retrieval of early words in a sentence to resolve the meaning of later words. Retrieval gave rise to a bilateral P300 response. Later work on ambiguity resolution at subcategorizing verbs (Mecklinger et al., 1995; Friederici et al., 1998) observed a parietally distributed positive response around 345 ms (P345), whose amplitude increased when the status of an ambiguous argument had to be revised from subject to object, and whose occurrence was tied to participants’ reading span. The possibility that the P345 during sentence processing indexes retrieval is in line with Phillips et al.’s (2005) report of an early positivity during argument–verb dependency resolution at a main verb. These sentence processing reports converge on work from outside the sentence processing domain: Polich et al. (1983) report a P300 during tone retrieval, whose latency was positively correlated with digit-span test scores. Ergen et al. (2012) found matching results for letter retrieval (for review, see Friedman and Johnson, 2000; Polich, 2007; Rugg and Curran, 2007). The even-shorter latency of the present positivity may be captured by previous proposals that argument–verb dependency resolution in verb-final languages may be initiated prior to the main verb (Friederici and Mecklinger, 1996; Aoshima et al., 2004; Phillips et al., 2005). This is compatible with Fiebach et al.’s (2001) findings of a pre-verbal positivity during the resolution of argument–verb dependencies in German sentences. Furthermore, this may explain why the TP-region dipole time course showed an effect in a time window earlier than predicted.

An alternative explanation of the early topographical correlation effect and the associated TP-region time course is that these do not resemble a P300, but a negative ERP component peaking around 400 ms (N400), classically obtained for increased semantic integration demands during sentence processing (Van Petten, 1993; Van Petten and Luka, 2012). Reduced N400 amplitude for the long as compared to the short argument–verb dependencies is predicted by anticipation-based parsing accounts (Konieczny and Döring, 2003; Levy, 2008), where cumulative lexical pre-activation decreases processing load at the subcategorizing verb (Van Petten, 1993; Hagoort et al., 2004). Regardless of the additional amount of information provided with the constituents intervening between argument and verb, an increased argument–verb distance opens a time window for comparably slow lexical-associative mechanisms (Konieczny, 2000; Spivey et al., 2002; Konieczny and Döring, 2003) or sentence-type-frequency-based mechanisms (Levy, 2008) to increase verb predictions. For three reasons, however, we consider this interpretation less likely: first, the cortical generators of the N400 are rarely found in the left TP-region, but rather involve middle temporal cortices (Simos et al., 1997; Johnson and Hamm, 2000; Silva-Pereyra et al., 2003; Maess et al., 2006) or a left lateralized network of middle and inferior temporal cortices (Halgren et al., 2002; for review see Lau et al., 2008). Second, the classical scalp distribution of the N400 during sentence processing involves bilateral parieto-occipital sensors, with a slight tilt to the right hemisphere (Kutas and Van Petten, 1994; Lau et al., 2008). The ERP component at which we observed a stronger fMRI–EEG correlation for the long as compared to the short argument–verb distances had, however, a clearly left lateralized posterior distribution. Third, the differential fMRI–EEG correlation for long as compared to short argument–verb dependencies occurred too early to index a lexical-semantic response.

In sum, it is most plausible that the short latency of the observed response is related to the pre-verbal initiation of argument retrieval (Fiebach et al., 2001; Phillips et al., 2005), triggered by a pre-head attachment mechanism as described in the active filler hypothesis (Clifton and Frazier, 1988; Frazier and Clifton, 1989). According to this hypothesis, arguments stored in working memory will be retrieved as soon as gap occurrence becomes clear. It is, however, impossible that a semantic N400 response to the subcategorizing verb occurs prior to the verb itself. Nevertheless, a temporal overlap between an increased gap-initiated retrieval-related P300 and a decreased verb-initiated lexical-activation-related N400 is possible for a later time window. This is a potential compromise between working memory-decay-based processing theories that predict increased processing difficulty with increasing dependency distance (Gibson, 1998, 2000; Lewis et al., 2006) and anticipation-based processing theories that predict decreased processing difficulty with increasing dependency distance (Konieczny and Döring, 2003; Levy, 2008).

### Late IFG activity: Argument reordering

The current fMRI data show increased activation in the left IFG for object-first as compared to subject-first argument orders, which has been reported in previous imaging work from Hebrew, German and Japanese (Ben-Shachar et al., 2003; Friederici et al., 2006; Kinno et al., 2008; Obleser et al., 2011), independent of working memory demands: the argument–verb distances in the object-first sentences used in these studies were identical to the argument–verb distances of their subject-first counterparts. Because of this, neither the increased processing difficulty nor the increased IFG brain activation in these studies can be attributed to working memory demands, but can rather be attributed to argument reordering demands. From a psycholinguistic perspective, this entails that argument order changes increase difficulty and brain activation over storage demands (cf. Lewis et al., 2006). Anticipation-based parsing accounts may capture the increased processing difficulty in the above studies by reduced verb predictability (cf. Konieczny, 2000; Levy, 2008). However, given imaging reports that the IFG plays a role in reordering during sentence processing (Ben-Shachar et al., 2003; Friederici et al., 2006; Kinno et al., 2008; Obleser et al., 2011) and sequencing outside of the sentence processing domain (Gerton et al., 2004; Clerget et al., 2011; Makuuchi et al., 2012), the proposal of an executive argument reordering mechanism is plausible from a cognitive-neuroscience perspective.

Considering that the topographical correlations showed a late left frontal positivity correlate of the IFG activity observed in the fMRI data, we suggest that our combined fMRI–EEG analysis provides evidence for a close relationship between reordering-related IFG activity and reordering-related late positive ERP components occurring around 600 ms (P600). While the late effect in the dipole time course of the IFG (see Figure 4A) is in line with this proposal, it is very unlikely that the IFG is the single generator of the P600: Service et al. (2007) have shown that P600 generators may span from bilateral middle temporal to left inferior frontal generators across participants. IFG activity can nevertheless account at least partly for the sensor-level time courses observed in the present data, and its involvement is directly implied by the source level time courses. While responses in the P600 range were first observed in response to syntactic violations (Osterhout and Holcomb, 1992, 1993; Friederici et al., 1993; Hagoort et al., 1993), later work found that a P600 response at subcategorizing verbs does also occur in ambiguous sentences. These do not contain violations, but instead require the revision of an initial interpretation (Friederici et al., 1998). More recently, Kaan et al. (2000) proposed to interpret the P600 as a general index of syntactic processing difficulty, with sentential complexity giving rise to an anterior scalp distribution and revision processes giving rise to a posterior scalp distribution (Kaan and Swaab, 2003).

While we suggested above that early posterior positivities rather reflect retrieval from working memory, our proposal that late anterior P600 effects may index argument reordering more specifically is compatible with Kaan and Swaab’s (2003) suggestion. Furthermore, the reordering proposal is in line with previous German data by Rösler et al. (1998), who report a P600 response for object-first as compared to subject-first argument orders, setting on before and continuing during the occurrence of the verb. Converging on these data, Friederici et al. (2002) report a fronto-centrally distributed P600 for object-first as compared to subject-first sentences at subcategorizing verbs. Explaining these previous effects and the current P600 effect in terms of decay-based sentence processing frameworks (Gibson, 1998, 2000; Lewis et al., 2006)–-without recurring to an executive reordering mechanism–-is tricky: out stimuli kept the argument–verb distance–-and thus the amount of decay an argument underwent–-constant across both subject-first and object-first sentences, ruling out an explanation of the P600 in terms of differential retrieval demands. From a functional-neuroimaging perspective, the brain data speak in favor of a reordering mechanism, potentially shared between various cognitive domains. While Levy’s (2008) sentence processing account may mirror this effect, we do not propose that any reordering-related or sequencing-related IFG activation is just a direct reflection of corpus frequencies.

The interpretation of the current results in terms of a dissociation of late reordering-related fronto-central P600 components and the early retrieval-related posterior positive ERP responses is in line with the results of Vos et al. (2001). In their ERP study, they compared object-first and subject-first argument orders at the subcategorizing verb. The researchers find an increased late frontal positivity for object-first as compared to subject-first argument orders only for low-span participants; for high-span participants, the authors find an early posterior positivity instead. This dissociation fits our results in that it may reflect low-span participants’ relative reliance on reordering processes (as indexed by the P600), whereas high-span participants may rely relatively stronger on their working memory capacity (as indexed by the early positivity).

## Conclusion

Based on combined fMRI–EEG results as well as evidence from the fMRI and EEG literature, we propose that working memory retrieval of arguments and argument reordering are core neurocognitive functions of argument–verb dependency resolution in sentence processing. During argument retrieval at the subcategorizing verb, the left TP-region supports initial argument retrieval, while later argument reordering is subserved by left IFG. The data and preliminary framework presented here generate testable hypotheses for both behavioral and neuroimaging studies; they demonstrate that joint fMRI–EEG analyses provide the explanatory power to reconcile models from cognitive neuropsychology and psycholinguistics with our increasing knowledge on human functional neuroanatomy.

## Conflict of Interest Statement

The authors declare that the research was conducted in the absence of any commercial or financial relationships that could be construed as a potential conflict of interest.
